# *Mycobacterium africanum* Is Associated with Patient Ethnicity in Ghana

**DOI:** 10.1371/journal.pntd.0003370

**Published:** 2015-01-08

**Authors:** Adwoa Asante-Poku, Dorothy Yeboah-Manu, Isaac Darko Otchere, Samuel Y. Aboagye, David Stucki, Jan Hattendorf, Sonia Borrell, Julia Feldmann, Emelia Danso, Sebastien Gagneux

**Affiliations:** 1 Bacteriology Department, Noguchi Memorial Institute for Medical Research, University of Ghana, Legon, Ghana; 2 Department of Medical Parasitology and Infection Biology, Swiss Tropical and Public Health Institute, Basel, Switzerland; 3 University of Basel, Basel, Switzerland; 4 Department of Epidemiology and Public Health, Swiss Tropical and Public Health Institute, Basel, Switzerland; University of Tennessee, United States of America

## Abstract

*Mycobacterium africanum* is a member of the *Mycobacterium tuberculosis* complex (MTBC) and an important cause of human tuberculosis in West Africa that is rarely observed elsewhere. Here we genotyped 613 MTBC clinical isolates from Ghana, and searched for associations between the different phylogenetic lineages of MTBC and patient variables. We found that 17.1% (105/613) of the MTBC isolates belonged to *M. africanum*, with the remaining belonging to *M. tuberculosis* sensu stricto. No *M. bovis* was identified in this sample. *M. africanum* was significantly more common in tuberculosis patients belonging to the Ewe ethnic group (adjusted odds ratio: 3.02; 95% confidence interval: 1.67–5.47, p<0.001). Stratifying our analysis by the two phylogenetic lineages of *M. africanum* (i.e. MTBC Lineages 5 and 6) revealed that this association was mainly driven by Lineage 5 (also known as *M. africanum* West Africa 1). Our findings suggest interactions between the genetic diversity of MTBC and human diversity, and offer a possible explanation for the geographical restriction of *M. africanum* to parts of West Africa.

## Author Summary

Tuberculosis remains one of the main global public health problems. Human tuberculosis is caused by bacteria known as the *Mycobacterium tuberculo*sis complex (MTBC). The MTBC includes a variant called *Mycobacterium africanum*, which causes up to half of all tuberculosis cases in West Africa. For reasons unknown, *M. africanum* does not occur in other parts of the world. To explore the possible reasons for this geographic restriction of *M. africanum*, we analysed a large collection of bacterial strains isolated from tuberculosis patients in Ghana. We genetically characterized these bacterial isolates and collected relevant socio-demographic and epidemiological data. We found tuberculosis patients infected with *M. africanum* were more likely to belong to the Ewe ethnic group, compared to patients carrying other MTBC bacteria. The Ewes are indigenous inhabitants of coastal regions in West Africa that have previously been shown to harbour a high prevalence of *M. africanum*. Our findings support the hypothesis that different variants of MTBC have adapted to different human populations, and offer a possible explanation for the geographical restriction of *M. africanum* to West Africa.

## Introduction

Tuberculosis (TB) remains the leading cause of adult death by a single infectious disease world-wide [1]. Despite the high mortality caused by TB, only 5% to 10% of infected immunocompetent individuals progress from initial infection to active disease [1]. In 2013, an estimated 9.0 million new cases and 1.5 million deaths due to TB occurred; with 30% of the global burden of TB occurring in Africa, an indication of the strong association with HIV/AIDS [1].

TB is caused by a group of closely related bacteria referred to as the *Mycobacterium tuberculosis* complex (MTBC). MTBC comprises *M. tuberculosis* sensu stricto and *M. africanum* which are the main agents of TB in humans, and several variants adapted to various domestic and wild mammal species, including *M. bovis*, *M. caprae, M. microti* and *M. pinnipedii* [2]. MTBC relevant to human disease has been classified into seven main phylogenetic lineages [3]–[4]: Lineages 1 to 4 together with Lineage 7 are collectively known as *M. tuberculosis* sensu stricto, whereas Lineage 5 and 6 are also known as *M. africanum* West Africa I and II, respectively [5].

Because MTBC harbours limited genetic diversity compared to other bacteria [6], for a long time the assumption was that host and environmental factors were the only relevant determinants driving the course of TB infection. However, recent studies have challenged this dogma. Indeed, experimental infection models have shown that MTBC strains differ in virulence and immunogenicity [7], and epidemiological studies have demonstrated that in addition to host and environmental factors, strain diversity contributes to the variable outcome of TB infection and disease in clinical settings [8].

The MTBC lineages adapted to humans exhibit a strong phylogeographic population structure [4]. This together with the finding that the MTBC most likely originated in Africa and accompanied human migrations over millennia [9] has led to the proposal that the different lineages of human-associated MTBC might have locally adapted to different human populations [10]. Support for this notion comes from the observation that in metropolitan settings, MTBC lineages tend to transmit preferentially among sympatric (as opposed to allopatric) host populations [11], and that this sympatric host-pathogen association is perturbed by HIV co-infection [12].

Previous work showed that in Ghana, human TB is caused by six out of the seven MTBC lineages, with 20% of all cases attributed to Lineages 5 and 6 [13] (i.e. *M. africanum* West-Africa I and West-Africa II, respectively). *M. africanum* is highly restricted to West-Africa for reasons unknown [5], [10]. One possibility could be that *M. africanum* has adapted to particular human populations in that region of the world. To address this possibility, we performed a retrospective molecular epidemiological study of MTBC in Southern Ghana. We combined bacterial genotyping with detailed demographic and epidemiological patient data and sought for associations between host factors and the main MTBC lineages prevailing in Ghana.

## Methods

### Ethics statement

All study protocols including oral and written consent format used for this study were approved by the Institutional Review Board (IRB) of the Noguchi Memorial Institute for Medical Research, Legon-Ghana (NMIMR; Federal wide Assurance number FWA00001824) and from the Ethikkommission Beider Basel (EKBB) in Basel, Switzerland. The standard procedure for sampling as outlined by the National Tuberculosis Program (NTP) for the routine management of TB in Ghana was used in the study. Written (in the case of literate participants) or oral (for illiterates) informed consent was sought from all participants before inclusion in the study. For minors (below sixteen years of age) consent was sought from their parents/guardians before enrolment into the study. In the case of minors between sixteen and eighteen years, in addition to parental consent, assent was sought from them before enrolment into the study. As per the guidelines of the IRB of NMIMR, information confidentiality was strictly adhered to. In addition, objectives and benefits of the study were explained to all participants.

### Study setting and patients' characteristics

The study was conducted from July 2007 to August 2011. All patients were diagnosed as sputum Acid-Fast-Bacilli-positive pulmonary TB cases by microscopy. The patients were recruited before treatment initiation from five main health facilities; Korle-Bu Teaching Hospital in the Greater Accra region, Agona Swedru Government Municipal Hospital, Winneba Government Hospital, St Gregory Catholic Clinic from the Central Region and Effia-Nkwanta Regional Hospital from Western Region of Ghana. Information on age, sex, nationality, ethnicity, employment status, previous history of TB, crowding, substance abuse and duration of symptoms were obtained from the patients with a structured questionnaire. Patients with missing information or culture-negative status were excluded from analysis. Ethnicity was classified in line with Ghana demographic data 2010 [14]. Patient origin was defined by place of residence.

### Isolation of mycobacterial species and genotyping

Sputum samples obtained were decontaminated using 5% oxalic acid [15] and inoculated on two pairs of Lowenstein Jensen (LJ) slants; one supplemented with 0.4% sodium pyruvate to enhance the isolation of *M. africanum* and *M. bovis*, and the other with glycerol for the growth of *M. tuberculosis* sensu stricto. The cultures were incubated at 37°C and were read weekly for growth for a maximal duration of 16 weeks. MTBC strains were identified by detection of insertion sequence IS*6110* as previously described [16]. Classification into the main phylogenetic lineages of MTBC was achieved by large sequence polymorphism typing identifying regions of difference (RD) [2] in a stepwise manner. Firstly, all isolates were screened for RD9. RD9-undeleted strains were further sub-typed for the “Cameroon” strain family (known to be most dominant Lineage 4 sub-lineage in Ghana) by screening for deletion of RD726 [11]. Isolates identified as RD9-deleted were subsequently sub-typed for Lineage 5 and 6 using RD711 and RD702 flanking primers, respectively. All lineages identified were confirmed by TaqMan real time PCR (TaqMan, Applied Bio systems, USA) using probes targeting lineage-specific SNPs as reported [17].

### Spoligotyping

All MTBC isolates were typed by spoligotyping [18]. This was performed according to the manufacturer's instructions, using commercially available kits (Isogen Bioscience BV Maarssen, The Netherlands). Spoligotyping patterns were defined according to SITVITWEB database [19] (http://www.pasteur-guadeloupe.fr:8081/SITVIT_ONLINE). SITVITWEB assigned shared types numbers were used whenever a spoligotyping pattern was found in the database while families and subfamilies were assigned based on the MIRU-VNTRplus database (http://www. miru-vntrplus.org) [20]. Shared types were defined as patterns common to at least two or more isolates. All patterns that could not be assigned were considered orphan spoligotypes.

### Data entry, management and analysis

Information from the structured questionnaire was double entered using Microsoft Access and validated to remove duplicates and data entry inconsistencies. Multivariable logistic regression models were used to compare patient characteristics associated with *M. africanum* compared to *M. tuberculosis* sensu stricto. All statistical analyses were performed in STATA 10.1 (Stata Corp., College Station, TX, USA).

## Results

### Tuberculosis patients and their characteristics

A total of 622 TB patients were included in this study. Age of patients ranged from 8 to 77 years with a median age of 35 years (Table 1). Overall, 208/622 (33.4%) were females with median age of 33 years; the remaining 414 (66.6%) were males with a median age of 36. Twenty-nine out of the 622 patients (4.6%) were children (age<16 years). Most patients originated from Greater Accra Region (325 cases, 52.3%), followed by 268 cases (43.1%) from Central Region with the remaining twenty-nine patients (4.6%) from Western Region of Ghana. Out of the 622 patients, 596 (95.8%) were Ghanaians, 21 (3.3%) were Liberians, 2 Togolese (0.3%) and 1 (0.2%) each of Nigerian, Ivorian and Gambian origin, respectively. Most of the patients were of Akan ethnicity (N = 427, 68.7%), followed by Ga (N = 104, 16.7%), Ewe (N = 71, 11.4%) with the remaining ethnicities accounting for 3.2% (N = 20). In terms of education, 436 patients (70.1%) were illiterates, 44 (7.1%) primary education, 132 (21.2%) had up to secondary education, and the remaining 10 (1.6%) tertiary education. More than half of the study population (N = 324, 52%) consumed alcohol on a regular basis, while only 44 (7%) smoked.

**Table 1:** Characteristics of patients included in the study.

Variable	N = 622	%
*Sex*		
Male	414	66.6
Female	208	33.4
*Age*		
Years 08–25	124	20.0
Years 26–40	282	45.3
Years 41–77	216	34.7
*Residency*		
Rural	117	18.8
Urban	505	81.2
*Region*		
Greater Accra	325	52.3
Central	268	43.1
Western	29	4.6
*Ethnicity*		
Akan	427	68.7
Ewe	71	11.4
Ga	104	16.7
Other	20	3.2
*Religion*		
Christian	564	90.7
Muslim	37	5.9
Pagan	21	3.4
*Level of Education*		
No education	436	70.1
Primary school	44	7.1
Secondary	132	21.2
Tertiary	10	1.6
*Alcohol Intake*		
Yes	324	52.1
No	298	47.9
*Smoking Status*		
Smokers	44	7.1
Non smokers	578	92.9
*Crowding(1-4 pers)*	195	31.4
(>5 pers)	427	68.6
*Occupation*		
Farmer	45	7.2
Others	577	92.8

### Prevalence of MTBC lineages and sub-lineages

MTBC isolates were obtained from all 622 TB patients. Upon genotyping, 9 of these (1.4%) produced ambiguous results and were thus excluded from further analysis. Hence, a total of 613 isolates were used for further analysis. Based on LSP and SNP typing, we identified six out of the seven human-associated MTBC lineages in our study sample (Table 2). The dominant lineages were Lineage 4 with 483 cases (78.8%), Lineage 5 (N = 86, 14.0%) and Lineage 6 (N = 19, 3.1%). Eleven isolates (1.8%) belonged to Lineage 1, 10 to Lineage 2 (includes Beijing; 1.6%), and the remaining 4 isolates to Lineage 3 (0.7%). Among the 483 Lineage 4 isolates, 313/483 (65.0%) belonged to the sub-lineage of Lineage 4 known as the ‘Cameroon family’. No *M. bovis* was identified in our sample.

**Table 2:** Genotyping profiles of 613 *M. tuberculosis* complex isolates from Ghana.

Species	SNP	RD9	RD726	RD711	RD702	Spoligotyping profile	Sub-lineage^a^	SIT	No	%
MTBss	L1	Undel	Undel	ND	ND	▪□□▪▪▪▪□□□▪▪▪▪▪▪▪▪▪▪▪▪▪▪▪▪▪▪□□□▪□▪▪▪▪▪▪▪▪▪▪	EAI	340	8	1.3
MTBss	L1	Undel	Undel	ND	ND	▪▪□▪▪▪▪▪▪▪▪▪▪▪▪▪▪▪▪▪▪▪▪▪▪▪▪▪□□□▪□□▪▪▪▪▪□▪▪▪	EAI		1	0.2
MTBss	L1	Undel	Undel	ND	ND	▪▪□▪▪▪▪▪▪▪▪▪▪▪▪▪▪▪▪▪▪▪▪▪▪▪▪▪□□□▪□▪▪▪▪▪▪▪▪▪▪	EAI	342	1	0.2
MTBss	L1	Undel	Undel	ND	ND	▪▪▪▪▪▪▪▪▪▪▪▪▪▪▪▪▪▪▪▪▪▪▪▪▪▪▪▪□□□▪□▪▪▪▪▪▪▪▪▪▪	EAI	236	1	0.2
MTBss	L2	Undel	Undel	ND	ND	□□□□□□□□□□□□□□□□□□□□□□□□□□□□□□□□□□▪▪▪▪▪▪▪▪▪	Beijing	1	10	1.6
MTBss	L3	Undel	Undel	ND	ND	▪□□▪□□□□□□□□▪▪▪▪▪▪▪▪▪▪□□□□□□□□□□□□▪▪▪▪▪▪▪▪▪	DEHLI/CAS		2	0.3
MTBss	L3	Undel	Undel	ND	ND	▪▪▪□□□□▪▪▪▪▪▪▪□▪▪▪▪▪▪□□□□□□□□□□□□□▪▪▪▪▪▪▪▪▪	DEHLI/CAS		1	0.2
MTBss	L3	Undel	Del	ND	ND	▪▪▪□□□□▪□▪▪▪▪▪▪▪▪▪▪▪▪▪□□□□□□□□□□□□▪▪▪▪▪▪▪▪▪	DEHLI/CAS	1092	1	0.2
MTBss	L4	Undel	Del	ND	ND	▪▪▪▪▪▪▪▪▪▪▪▪▪▪▪▪▪▪▪▪▪▪□□□▪▪▪▪▪▪▪□□□□▪▪▪▪▪▪▪	Cameroon	61	226	36.8
MTBss	L4	Undel	Del	ND	ND	▪▪▪▪▪▪▪▪▪▪▪▪▪▪▪▪▪▪▪▪▪▪□□□▪▪▪□□▪▪□□□□▪▪▪▪▪▪▪	Cameroon	772	20	3.2
MTBss	L4	Undel	Del	ND	ND	▪▪▪▪▪▪▪▪▪▪▪▪▪▪□▪▪▪▪▪▪▪□□□▪▪▪▪▪▪▪□□□□▪▪▪▪▪▪▪	Cameroon	115	7	1.1
MTBss	L4	Undel	Del	ND	ND	▪▪▪▪▪▪▪▪▪▪▪▪▪▪▪▪▪▪▪▪▪▪□□□▪▪▪▪▪▪▪□□□□▪▪▪▪□▪▪	Cameroon	838	3	0.4
MTBss	L4	Undel	Del	ND	ND	▪▪▪▪▪▪▪▪▪▪▪▪▪□▪▪▪▪▪▪▪▪□□□▪▪▪▪▪▪▪□□□□▪▪▪▪▪▪▪	Cameroon		26	4.2
MTBss	L4	Undel	Del	ND	ND	▪▪▪▪▪▪▪▪▪▪▪▪▪▪▪▪▪▪▪▪▪▪□□□▪▪▪▪▪▪▪□□□□▪▪▪□▪▪▪	Cameroon		1	0.2
MTBss	L4	Undel	Del	ND	ND	▪▪▪▪▪▪▪□▪▪▪▪▪▪□▪▪▪▪▪▪▪□□□▪▪▪▪▪▪▪□□□□▪▪□▪▪□▪	Cameroon		1	0.2
MTBss	L4	Undel	Del	ND	ND	▪▪▪▪▪▪▪▪▪▪▪▪□▪□▪▪▪▪▪▪▪□□□▪▪▪▪▪▪▪□□□□▪▪▪▪▪▪▪	Cameroon		2	0.3
MTBss	L4	Undel	Del	ND	ND	▪▪▪▪▪▪▪▪▪▪▪▪▪▪▪▪▪▪▪▪▪▪□□□□▪▪▪▪▪▪□□□□▪▪▪▪▪▪▪	Cameroon	1141	1	0.2
MTBss	L4	Undel	Del	ND	ND	▪▪▪▪▪▪▪▪▪▪▪▪▪▪▪▪▪▪▪▪▪▪□□□▪▪▪▪▪▪▪□□□□▪▪▪□▪▪▪	Cameroon	403	1	0.2
MTBss	L4	Undel	Del	ND	ND	▪▪▪▪▪▪▪▪▪▪▪▪▪▪▪▪▪▪▪▪▪□□□□▪▪▪▪▪▪▪□□□□▪▪▪▪▪▪▪	Cameroon		2	0.3
MTBss	L4	Undel	Del	ND	ND	▪▪▪▪▪▪▪▪▪▪▪▪□▪▪▪▪▪▪▪▪▪□□□▪▪▪□□▪▪□□□□▪▪▪▪▪▪▪	Cameroon		2	0.3
MTBss	L4	Undel	Del	ND	ND	▪▪▪▪▪▪▪▪▪▪▪▪□▪▪▪▪▪▪▪▪▪□□□□□▪▪▪▪▪□□□□▪▪▪▪▪▪▪	Cameroon		3	0.4
MTBss	L4	Undel	Del	ND	ND	▪▪▪▪▪▪▪▪▪▪▪▪▪▪□▪▪▪▪▪▪▪□□□▪▪▪▪▪▪▪□□□□▪▪□▪▪□▪	Cameroon		2	0.3
MTBss	L4	Undel	Del	ND	ND	▪▪▪▪▪▪▪▪▪▪▪▪▪▪▪▪▪▪▪▪▪▪□□□▪▪▪□▪▪▪□□□□▪▪▪▪▪▪▪	Cameroon		1	0.2
MTBss	L4	Undel	Del	ND	ND	▪▪▪▪▪▪▪▪▪▪▪▪▪▪▪▪▪▪▪▪▪▪□□□□□□▪▪▪▪□□□□▪▪▪▪▪▪▪	Cameroon		1	0.2
MTBss	L4	Undel	Del	ND	ND	▪▪▪▪□▪▪▪▪▪▪▪▪▪▪▪▪▪▪▪▪▪□□□▪▪▪□□▪▪□□□□▪▪▪▪▪▪▪	Cameroon		1	0.2
MTBss	L4	Undel	Del	ND	ND	▪▪▪□□□□□□□□□□□□□□□□□□□□□□□□□□□□□□□□□▪▪▪▪▪▪▪	Cameroon		2	0.3
MTBss	L4	Undel	Del	ND	ND	▪▪▪▪▪▪▪▪▪▪▪▪▪▪□▪▪▪▪▪▪□□□□▪▪▪▪▪▪▪□□□□▪▪□▪▪□▪	Cameroon		3	0.4
MTBss	L4	Undel	Del	ND	ND	▪▪▪▪▪▪▪▪▪▪▪▪▪▪▪▪▪▪▪▪▪▪□□□▪▪▪□▪▪▪□□□□▪▪▪▪▪▪▪	Cameroon		1	0.2
MTBss	L4	Undel	Del	ND	ND	▪□□▪▪▪▪▪▪▪▪▪▪▪▪▪▪▪▪▪▪▪□□□▪▪▪▪▪▪▪□□□□▪▪▪▪▪▪▪	Cameroon		2	0.3
MTBss	L4	Undel	Del	ND	ND	▪▪▪▪▪▪▪▪▪▪▪▪▪▪▪▪▪▪▪▪▪▪□□□□□□□▪▪▪□□□□▪▪▪▪▪▪▪	Cameroon		3	0.4
MTBss	L4	Undel	Del	ND	ND	▪▪▪▪▪▪▪▪▪▪▪▪▪▪▪▪▪▪▪▪▪▪□□□▪▪▪▪▪▪▪□□□□▪▪□□▪□▪	Cameroon		1	0.2
MTBss	L4	Undel	Del	ND	ND	▪▪▪▪▪▪▪▪▪▪▪▪▪▪▪▪▪▪▪▪▪▪□□□▪▪▪▪▪▪▪□□□□▪□□□□□□	Cameroon		1	0.2
MTBss	L4	Undel	Undel	ND	ND	▪▪▪▪▪▪▪▪▪▪▪▪▪▪▪▪▪▪▪▪▪▪▪▪▪▪▪▪▪▪▪▪□□□□▪▪▪▪▪▪▪	Ghana	53	26	4.2
MTBss	L4	Undel	Undel	ND	ND	▪▪▪▪▪▪▪▪▪▪▪▪▪▪▪▪▪▪▪▪▪▪▪▪▪▪▪▪▪▪▪▪□□□□▪□□▪▪▪▪	Ghana	65	4	0.7
MTBss	L4	Undel	Undel	ND	ND	▪▪▪▪▪▪▪▪▪▪▪▪□▪▪▪▪▪▪▪▪□▪▪▪▪▪▪▪▪▪▪□□□□▪▪▪▪▪▪▪	Ghana	504	7	1.1
MTBss	L4	Undel	Undel	ND	ND	▪▪▪▪▪▪▪▪▪▪▪▪▪▪□▪▪▪▪▪▪▪▪▪▪▪▪▪▪▪▪▪□□□□▪▪▪▪▪▪▪	Ghana	118	12	1.9
MTBss	L4	Undel	Undel	ND	ND	▪□□▪▪▪▪▪▪▪▪▪▪▪▪▪▪▪▪▪▪▪▪▪▪▪▪▪▪▪▪▪□□□□▪▪▪▪▪▪▪	Ghana	804	1	0.2
MTBss	L4	Undel	Undel	ND	ND	▪▪▪▪▪▪▪▪▪▪▪▪▪▪▪▪▪▪▪▪▪▪▪▪▪▪▪▪□▪▪▪□□□□▪▪▪▪▪▪▪	Ghana	462	4	0.7
MTBss	L4	Undel	Undel	ND	ND	▪▪▪▪▪▪▪▪▪▪▪▪▪▪▪▪▪▪▪▪▪▪□▪▪▪▪▪▪▪▪▪□□□□▪▪▪▪▪▪▪	Ghana	44	1	0.2
MTBss	L4	Undel	Undel	ND	ND	▪▪▪▪▪▪▪▪▪▪▪▪▪▪▪▪▪▪▪▪▪□▪▪▪▪▪▪▪▪▪▪□□□□▪▪▪▪▪▪▪	Ghana	86	12	1.9
MTBss	L4	Undel	Undel	ND	ND	▪▪▪▪▪▪▪▪▪▪▪▪▪▪▪▪▪▪▪▪▪▪▪▪▪▪▪▪▪□▪▪□□□□▪▪▪▪▪▪▪	Ghana	167	1	0.2
MTBss	L4	Undel	Undel	ND	ND	▪▪▪▪▪▪▪▪▪▪▪▪▪▪▪▪▪▪▪▪▪▪▪□▪▪▪▪▪▪▪▪□□□□▪▪▪▪▪▪▪	Ghana	373	1	0.2
MTBss	L4	Undel	Undel	ND	ND	▪▪▪▪▪▪▪▪▪▪▪▪▪□▪▪▪▪▪▪▪▪▪▪▪▪▪▪▪▪▪▪□□□□▪▪▪▪▪▪▪	Ghana	393	1	0.2
MTBss	L4	Undel	Undel	ND	ND	□□□□▪▪▪▪▪▪▪▪▪▪▪▪▪▪▪▪▪▪▪▪▪▪▪▪▪▪▪▪□□□□▪▪▪▪▪▪▪	Ghana	272	1	0.2
MTBss	L4	Undel	Undel	ND	ND	▪▪▪▪▪▪▪▪▪▪▪▪▪▪▪▪▪▪▪▪▪▪▪▪▪▪▪▪▪▪▪▪□□□□▪▪□▪▪□▪	Ghana		4	0.7
MTBss	L4	Undel	Undel	ND	ND	▪□□▪▪▪▪▪▪▪▪▪▪▪▪▪▪▪▪▪▪▪▪▪▪▪□□□□□▪□□□□▪▪▪▪▪▪▪	Haarlem	1652	4	0.7
MTBss	L4	Undel	Undel	ND	ND	▪▪▪▪▪▪▪▪▪▪▪▪▪▪▪▪▪▪▪▪▪▪▪▪▪▪□□□□□□□□□□▪▪▪▪▪▪▪	Haarlem	1498	6	0.9
MTBss	L4	Undel	Undel	ND	ND	▪▪▪▪▪▪▪▪▪▪▪▪▪▪▪▪▪▪▪▪▪▪▪▪▪▪▪▪▪▪□▪□□□□▪▪▪▪▪▪▪	Haarlem	50	15	2.4
MTBss	L4	Undel	Undel	ND	ND	▪▪▪▪▪▪▪▪▪▪▪▪▪▪▪▪▪▪▪▪▪▪▪□▪□□□□□□▪□□□□▪▪▪▪▪▪▪	Haarlem	45	2	0.3
MTBss	L4	Undel	Undel	ND	ND	▪□□▪▪▪▪▪▪▪▪▪▪▪▪▪▪▪▪▪▪▪▪▪▪▪▪▪▪▪□▪□□□□▪▪▪▪▪▪▪	Haarlem	655	3	0.4
MTBss	L4	Undel	Undel	ND	ND	▪▪▪▪▪▪▪▪▪▪▪▪▪▪▪▪▪▪▪▪▪▪▪▪▪▪□□□□□▪□□□□▪▪▪▪▪▪▪	Haarlem	47	2	0.3
MTBss	L4	Undel	Undel	ND	ND	▪▪▪▪▪▪▪▪▪▪▪▪▪▪▪▪▪▪▪▪▪▪▪▪▪▪□□□□□▪□□□□▪▪▪□▪▪▪	Haarlem	62	2	0.3
MTBss	L4	Undel	Undel	ND	ND	▪▪▪▪▪▪▪▪▪▪▪▪▪▪▪▪▪▪▪▪▪▪▪▪▪▪▪□□□□□□□□□▪▪□▪▪□▪	Haarlem		2	0.3
MTBss	L4	Undel	Undel	ND	ND	▪▪▪▪▪▪▪▪▪▪▪▪▪▪▪▪▪▪▪▪▪▪▪▪▪▪□□□□□▪□□□□▪▪▪□□▪▪	Haarlem		1	0.2
MTBss	L4	Undel	Undel	ND	ND	▪▪▪▪▪▪▪▪▪▪▪▪▪▪▪▪▪▪▪▪□□□□□□▪▪□▪▪▪□□□□▪▪▪▪▪▪▪	LAM	306	1	0.2
MTBss	L4	Undel	Undel	ND	ND	▪▪▪▪▪▪▪□□□□□□▪▪▪▪▪▪▪□□□□▪▪▪▪▪▪▪▪□□□□▪▪▪▪▪▪▪	LAM		1	0.2
MTBss	L4	Undel	Undel	ND	ND	▪▪▪▪▪▪▪▪▪▪▪▪▪▪▪▪▪▪▪▪□□□□▪▪▪▪▪▪▪▪□□□□▪▪▪▪▪▪▪	LAM	42	2	0.3
MTBss	L4	Undel	Undel	ND	ND	▪▪▪▪▪▪▪▪□□□▪▪▪▪▪▪▪▪▪□□□□▪▪▪▪▪▪▪▪□□□□▪▪▪▪▪▪▪	LAM	33	1	0.2
MTBss	L4	Undel	Undel	ND	ND	▪▪▪□□□□□□□□▪▪▪▪▪▪□▪▪□□□□▪▪▪▪▪▪▪▪□□□□▪▪□▪▪▪▪		70	7	1.1
MTBss	L4	Undel	Undel	ND	ND	▪▪▪▪▪▪▪▪▪▪▪▪▪▪▪▪▪▪▪▪▪▪▪▪▪▪▪▪▪▪□▪□□□□▪▪□□□□▪	Uganda I		2	0.3
MTBss	L4	Undel	Undel	ND	ND	▪▪▪▪▪▪▪▪▪▪▪▪▪▪▪▪▪▪▪▪▪▪▪▪▪▪▪▪▪▪▪▪□□□□▪▪▪□▪▪▪	Uganda I	52	4	0.7
MTBss	L4	Undel	Undel	ND	ND	▪▪▪▪▪▪▪▪▪▪▪▪▪▪▪▪▪▪▪▪▪▪▪▪▪▪▪▪▪▪▪▪□□□□▪▪□□□□▪	Uganda I	244	1	0.20.4
MTBss	L4	Undel	Undel	ND	ND	▪▪▪□▪▪▪▪▪▪▪▪▪▪▪▪▪▪▪▪▪▪▪▪▪▪▪▪▪▪▪▪□□□□▪▪▪□▪▪▪	Uganda I	848	3	
MTBss	L4	Undel	Undel	ND	ND	▪▪▪▪▪▪▪▪▪▪▪▪▪▪▪▪▪▪▪▪▪▪▪▪▪▪▪▪▪▪▪▪□□□□▪▪▪□□▪▪	Uganda I		2	0.3
MTBss	L4	Undel	Undel	ND	ND	▪▪▪▪▪▪▪▪▪▪▪▪▪▪▪▪▪▪▪▪▪▪▪▪▪▪▪▪▪▪▪▪□□□□▪▪□□▪□▪	Uganda I	78	1	0.2
MTBss	L4	Undel	Undel	ND	ND	▪▪▪▪▪▪▪▪▪▪▪▪▪▪▪▪▪▪▪▪▪▪▪▪▪▪▪▪▪▪□▪□□□□▪▪□□□□▪	Uganda I		1	0.2
MTBss	L4	Undel	Undel	ND	ND	□□□□□□□□□□□□□□□□□□□□□□□□▪▪▪▪▪▪▪▪□□□□▪▪□□□□▪	Uganda I	125	1	0.2
MTBss	L4	Undel	Undel	ND	ND	▪▪▪▪▪▪▪▪▪▪▪▪▪▪▪▪▪▪▪▪▪▪▪▪▪▪▪▪▪▪▪▪□□□□▪▪▪□□□□	Uganda II	51	2	0.3
MTBss	L4	Undel	Undel	ND	ND	▪▪▪▪▪▪▪▪▪□□▪□□▪▪▪▪▪▪▪□▪▪▪▪▪▪▪▪▪▪□□□□▪▪□□▪□▪	Uganda II		2	0.3
MTBss	L4	Undel	Undel	ND	ND	▪▪▪▪▪▪▪▪▪▪□□□□□□▪▪▪▪▪□▪▪▪▪▪▪▪▪□▪□□□□▪▪▪▪▪▪▪	Uganda II		3	0.4
MTBss	L4	Undel	Undel	ND	ND	▪▪▪▪▪▪▪□□□□▪▪▪▪▪▪▪▪▪▪▪▪▪▪▪▪▪▪▪▪▪□□□□▪▪▪▪▪▪▪	S	1223	2	0.3
MTBss	L4	Undel	Undel	ND	ND	▪□▪▪▪▪▪□□▪▪▪▪▪▪▪▪▪▪▪▪▪▪▪▪▪▪▪▪▪▪▪□□□□▪▪▪▪▪▪▪	S	1211	2	0.3
MTBss	L4	Undel	Undel	ND	ND	▪▪▪▪▪▪▪▪▪▪▪▪▪▪▪▪▪□▪▪▪▪▪▪▪▪▪▪▪▪▪▪□□□□▪▪▪▪▪▪▪	X	119	2	0.3
MTBss	L4	Undel	Undel	ND	ND	▪▪▪□□□□□□□□□▪▪▪▪▪□▪▪▪▪▪▪▪▪▪▪▪▪▪▪□□□□▪▪▪□□□□		200	7	1.1
MTBss	L4	Undel	Undel	ND	ND	▪▪□▪▪▪▪□□□□□▪□▪▪▪▪▪▪□□□□▪▪▪▪▪▪▪▪□□□□▪▪▪▪▪▪▪			2	0.3
MTBss	L4	Undel	Undel	ND	ND	▪▪▪▪▪▪▪▪□□□□▪□▪▪▪▪▪▪▪▪▪▪▪▪▪▪▪▪▪▪□□□□▪▪▪▪▪▪▪			2	0.3
MTBss	L4	Undel	Undel	ND	ND	▪□□▪▪▪▪▪▪▪▪▪▪▪□▪▪▪▪▪▪▪▪▪▪▪▪▪▪▪□▪□□□□▪▪▪▪▪▪▪			1	0.2
MTBss	L4	Undel	Undel	ND	ND	▪▪▪□□□□□□□▪□▪▪▪□▪▪▪▪▪▪▪▪▪▪▪▪▪▪▪▪□□□□▪▪▪▪▪▪▪			1	0.2
MTBss	L4	Undel	Undel	ND	ND	▪▪▪□□□□□□□▪▪▪▪□▪▪▪▪▪▪▪▪▪▪▪▪▪▪▪▪▪□□□□▪▪▪□□□□			4	0.7
MTBss	L4	Undel	Undel	ND	ND	▪▪▪▪▪▪▪▪▪▪▪▪▪▪▪▪▪□□▪▪▪▪▪▪▪▪▪▪▪▪▪□□□□▪▪▪▪▪▪▪			1	0.2
Mafric	L5	Del	ND	Del	Undel	▪▪▪▪▪▪▪▪□□□□□▪▪▪▪▪▪▪▪□□□□▪▪▪▪▪▪▪▪▪▪▪□□□▪▪▪▪	WA I	331	17	2.8
Mafric	L5	Del	ND	Del	Undel	▪□▪▪▪▪▪▪□□□□□▪▪□▪▪▪▪▪□□▪□▪▪▪▪▪▪▪▪▪▪▪□□□▪▪▪▪	WA I		1	0.2
Mafric	L5	Del	ND	Del	Undel	▪□▪▪▪▪▪▪□□□□□▪▪□▪▪▪▪▪□□□□□▪□□□□□□□□□□□□□▪▪▪	WA I		1	0.2
Mafric	L5	Del	ND	Del	Undel	▪▪▪▪▪▪▪▪□□□□□□□▪▪▪▪▪▪□□□□▪▪▪▪▪▪▪▪▪▪▪□□□▪▪▪▪	WA I		1	0.2
Mafric	L5	Del	ND	Del	Undel	▪□▪▪▪▪▪▪□□□□□□▪▪▪▪▪▪▪□□□□▪▪▪▪▪▪▪▪▪▪▪□□□▪▪▪▪	WA I	319	16	2.6
Mafric	L5	Del	ND	Del	Undel	▪▪▪▪▪▪▪▪□□□□□□▪▪▪▪▪▪▪▪▪▪▪▪▪▪▪▪▪▪▪▪▪▪□□□▪▪▪▪	WA I	438	9	1.5
Mafric	L5	Del	ND	Del	Undel	▪□▪▪▪▪▪▪□□□□□□▪▪▪▪▪▪▪□□□□▪▪▪▪▪▪▪▪▪▪▪□□□▪▪▪▪	WA I	860	1	0.2
Mafric	L5	Del	ND	Del	Undel	▪▪▪▪▪▪▪▪□□□□□▪▪▪▪▪▪▪▪□□□□□▪□□□□□□□□□□□□□▪▪▪	WA I	1592	2	0.3
Mafric	L5	Del	ND	Del	Undel	▪▪▪▪▪▪▪□□□□□▪▪▪▪□□□□□□□□▪▪□□□□□▪▪▪▪▪□□□▪▪▪▪	WA I		1	0.2
Mafric	L5	Del	ND	Del	Undel	▪▪▪▪▪▪▪▪▪□▪▪▪▪▪□▪▪▪▪□□□□▪▪▪▪▪▪▪▪▪▪▪▪□□□▪▪□▪	WA I		1	0.2
Mafric	L5	Del	ND	Del	Undel	▪▪▪▪▪▪▪□□□□▪▪▪▪▪▪▪▪▪□□▪□▪▪▪▪▪▪▪▪▪▪▪▪□□□▪▪□▪	WA I		1	0.2
Mafric	L5	Del	ND	Del	Undel	▪□▪▪▪▪▪▪□□□□□▪▪▪▪▪▪▪▪□□□□□▪▪▪▪▪▪▪▪▪▪□□□▪▪▪▪	WA I		3	0.4
Mafric	L5	Del	ND	Del	Undel	▪▪▪▪▪▪▪▪□□□□□▪▪□▪▪▪▪□□□□□□▪□▪▪▪□□□▪▪□□□□▪□▪	WA I		1	0.2

a Sub-lineage as defined by the MIRU-VNTRplus database, Undel = not deleted, Del = deleted, ND = Not done.

All isolates were further sub-typed using spoligotyping (Table 2). We detected a total of 117 spoligotypes, 485/613 isolates (79%) had previously defined shared type number (SIT). The remaining 128 isolates could not be defined by the SITVIT database and thus were defined as ‘orphan’. In addition to Cameroon sub-lineage, seven additional sub-lineages were identified among Lineage 4 based on spoligotyping; Ghana (N = 75, 15.5%), Haarlem (N = 37, 7.7%), Uganda I (N = 15, 3.1%), Uganda II (N = 7, 1.4%), LAM (N = 5, 1.0%), S (N = 4 (0.8%), and X (N = 2, 0.4%).

### Association between MTBC lineages and patient characteristics

Table 3 illustrates the association of socio demographic and behavioural factors with the main MTBC lineages present in our study sample. Using multivariable logistic regression model analysis, we found that individuals of Ewe ethnicity were significantly more likely to present with TB caused by *M. africanum* as opposed to *M. tuberculosis* sensu stricto irrespective of their place of residence (adjusted odds ratio (adjOR) = 3.02; 95% confidence interval (CI): 1.67–5.47, P<0.001) (Table 3, S1 Fig.). This association was independent from other risk factors. Moreover, we found TB caused by *M. africanum* to be associated with smoking (adjOR = 2.02; 95% CI: 0.95–4.27) when compared to *M. tuberculosis* sensu stricto. However, this association was only borderline statistically significant (P = 0.07). No significant associations between MTBC lineages and other patient variables were found. Because *M. africanum* comprises two phylogenetic distinct lineages (i.e. MTBC Lineages 5 and 6), we performed a stratified analysis by lineage. Using multivariate logistic regression model analysis, we observed a significant association between Ewe ethnicity and Lineage 5 (adjOR)  = 2.79; 95% CI: 1.47–5.29, P<0.001). This association was independent from other risk factors (Table 4). Interestingly, based on univariate analysis, we also saw an association between Ewe ethnicity and Lineage 6 (adjOR = 4.03; 95% CI: 1.33–10.85). However, because of the limited number of Lineage 6 isolates (n = 18) multivariate analyses could not be performed to confirm the independence of this association.

**Table 3:** Risk factors for TB caused by *M. africanum* compared to *M. tuberculosis* sensu stricto.

*Risk factor*	*%(n) Mafr*	*%(n) MTBss*	*OR (95%CI)*	*adjOR (95%CI)^a^*
	(n = 102)	(n = 511)		
Sex (male)	68% (69)	66% (338)	0.93 (0.59–1.47)	
Age category				
years 08–25	17% (17)	21% (105)	reference	
years 26–40	53% (54)	44% (223)	1.50 (0.83–2.70)	
years 41–77	30% (31)	36% (183)	1.05 (0.55–1.98)	
Rural residency	20% (20)	18% (93)	1.10 (0.64–1.88)	
Region				
Accra	55% (56)	52% (267)	reference	reference
Central	42% (43)	43% (218)	0.94 (0.61–1.45)	0.97 (0.60–1.56)
Western	3% (3)	5% (26)	0.55 (0.16–1.88)	0.44 (0.12–1.63)
Ethnicity				
Akan	58% (59)	71% (359)	reference	reference
Ewe	23% (23)	9% (48)	2.91 (1.65–5.14)^*^	3.02 (1.67–5.47)^*^
Ga	15% (15)	17% (89)	1.03 (0.56–1.89)	0.97 (0.51–1.83)
other	5% (5)	3% (15)	2.03 (0.71–5.79)	2.35 (0.77–7.13)
Religion				
Christian	92% (94)	90% (462)	reference	
Muslim	7% (7)	6% (29)	1.18 (0.50–2.79)	
Pagan	1% (1)	4% (20)	0.25 (0.03–1.85)	
Educational level				
No education	74% (75)	70% (356)	reference	
Primary school	6% (6)	7% (38)	0.75 (0.30–1.83)	
Secondary	21% (21)	23% (117)	0.85 (0.50–1.44)	
Alcohol	57% (58)	52% (263)	1.23 (0.81–1.90)	
Smoking	11% (11)	6% (32)	1.81 (0.88–3.72)	2.02 (0.95–4.27)^†^
Crowding (>5 pers)^b^	63% (64)	70% (359)	0.71 (0.45–1.10)	
Occupation farmer	9% (9)	7% (35)	1.32 (0.61–2.83)	

**Table 4:** Risk factors for Risk factor for TB caused by Lineage 5 compared to *M. tuberculosis* sensu stricto.

Risk factor	%(n) Lineage 5	%(n) MTBss	OR (95%CI)	adjOR (95%CI)^a^
	(n = 84)	(n = 511)		
Sex (male)	59% (58)	66% (338)	1.41 (0.69–1.88)	
Age category				
years 08–25	18% (15)	21% (105)	reference	
years 26–40	51% (43)	43% (223)	1.35 (0.72–2.54)	
years 41–77	31% (26)	36% (183)	0.99 (0.5–1.96)	
Rural residency	19% (16)	18% (93)	1.06 (0.59–1.91)	
Region				
Accra	54% (45)	52% (267)	reference	
Central	42% (36)	43% (218)	0.98 (0.61–1.57)	
Western	4% (3)	5% (26)	0.68 (0.2–2.36)	
Ethnicity				
Akan	61% (51)	70% (359)	reference	reference
Ewe	20% (17)	9% (48)	2.49 (1.33–4.66)**	2.79 (1.47–5.29)**
Ga	14% (12)	17% (89)	0.95 (0.49–1.86)	0.85 (0.43–1.69)
other	5% (4)	3% (15)	1.88 (0.6–5.88)	1.64 (0.53–5.34)
Religion				
Christian	93% (78)	90% (462)	reference	
Muslim	6% (5)	6% (29)	1.02 (0.38–2.72)	
Pagan	1% (1)	4% (20)	0.29 (0.04–2.24)	
Educational level				
No education	70% (59)	70% (356)	reference	
Primary school	7% (6)	7% (38)	0.95 (0.39–2.35)	
Secondary +	23% (19)	23% (117)	0.98 (0.56–1.71)	
Alcohol	62% (52)	52% (263)	1.53 (0.95–2.45)^†^	1.62 (0.99–2.68)^†^
Smoking	11% (9)	6% (32)	1.8 (0.82–3.91)	1.54 (0.68–3.50)
Crowding (>5 pers)^c^	63% (53)	70% (359)	0.72 (0.44–1.16)	
Occupation farmer	11% (9)	7% (35)	0.61 (0.28–1.32)	0.64 (0.29–1.45)

## Discussion

Our retrospective molecular epidemiological investigation of MTBC clinical isolates from Southern Ghana revealed that i) the Cameroon sub-lineage of Lineage 4 is the dominant cause of human TB in this region, ii) 17.1% of human TB is caused by *M. africanum*, iii) TB patients infected with *M. africanum* were more likely to smoke, and iv) to belong to the Ewe ethnic group.

Our finding that the Cameroon sub-lineage causes 65% of human TB in Ghana confirms our previous report from Ghana [13], and is in agreement with findings from neighbouring countries. In particular, the Cameroon sub-lineage was previously found to cause 40% of human TB in Cameroon [21], 45% in Nigeria [22] and 33% in Chad [23]. The reasons for the success of this sub-lineage in this region of Africa are unclear but could be due to a founder effect and/or particularly high fitness in the corresponding patient populations. Similarly, other successful sub-lineages of Lineage 4 have been observed in other regions of Africa, including Uganda [24] and Zimbabwe [25].

We found that in Ghana, *M. africanum* still accounts for 17.1% of all human TB, which is similar to the prevalence we reported several years ago [13]. This is in contrast to a study in Cameroon [21] that indicated a sharp decrease in TB caused by *M. africanum* during the last decades. A potential explanation for the decline of *M. africanum* in some West African countries includes possible out-competition by *M. tuberculosis*, as *M. africanum* has been associated with reduced virulence in animal models [26]–[27], and a longer latency and a slower rate of progression to active disease in humans [28]. Of note, our finding that smoking was associated with infection by *M. africanum* as opposed to *M. tuberculosis* sensu stricto is consistent with the notion that *M. africanum* might be less virulent in immunocompetent hosts [7]. This notion is also supported by a previous study in the Gambia reporting a significant association between *M. africanum* West Africa II and HIV co-infection [29]. However, no such association was found between *M. africanum* West Africa I and II in Ghana [30]. Because information on HIV status was not available for the present study, we could not explore this question here. Taken together, there is a need for further investigation to ascertain why *M. africanum* is declining in some regions of West Africa, but not in Ghana, and whether this phenomenon can be attributed to differences in virulence and/or other factors.

One reason for why the prevalence of *M. africanum* might be more stable in Ghana than in some other countries is that this bacterial lineage might be particularly well adapted to (some) human populations in Ghana. Our finding that *M. africanum* was independently associated with Ewe ethnicity supports this possibility. Moreover, this association was largely driven by Lineage 5, and not the result of a single outbreak as the spoligotyping patterns among *M. africanum* isolates from Ewe patients were diverse (Table 5). From available data, we know that *M. africanum*, in particular Lineage 5 is prevalent in countries around the Gulf of Guinea [13], [31], and particularly frequent in Benin and Ghana [13], [32], two countries with large Ewe populations [33]. The Ewe speaking ethnic group traditionally forms part of the Gbe language family which includes the Fons of Benin, the Aja of Togo and the Phla-phera of western Nigeria [33], [34]. Although the Ewe, Fons, Aja and phla-phera are different dialects of the same Gbe language family, members of theses individual groups are interrelated [33], [34]. Together they constitute the indigenous inhabitants of coastal West Africa.

**Table 5:** Spoligotyping profiles of *M. africanum* isolates from patients of Ewe ethnicity.

Species	SNP	RD726	RD711	RD702	Spoligotyping profile	Sub-lineage^a^	SIT	No	%
Mafric	L5	ND	Del	Undel	▪▪▪▪▪▪▪□□□□□▪▪▪▪□□□□□□□□▪▪□□□□□▪▪▪▪▪□□□▪▪▪▪	WA I		2	8.8
Mafric	L5	ND	Del	Undel	▪▪▪▪▪▪▪□□□□□□□▪▪▪▪▪▪▪▪▪▪▪▪▪▪▪▪▪▪▪▪▪▪□□□▪▪▪▪	WA I	438	5	21.7
Mafric	L5	ND	Del	Undel	▪▪▪▪▪▪▪▪□□□□□▪▪▪▪▪▪▪▪□□□□▪▪▪▪▪▪▪▪▪▪▪□□□▪▪▪▪	WA I	331	7	30.4
Mafric	L5	ND	Del	Undel	▪□▪▪▪▪▪▪□□□□□▪▪□▪▪▪▪▪□□▪□▪▪▪▪▪▪▪▪▪▪▪□□□▪▪▪▪	WA I	Orphan	1	4.3
Mafric	L5	ND	Del	Undel	▪□▪▪▪▪▪▪□□□□□□▪▪▪▪▪▪▪□□□□▪▪▪▪▪▪▪▪▪▪▪□□□▪▪▪▪	WA I	319	2	8.8
Mafric	L5	ND	Del	Undel	▪▪▪▪▪▪▪▪□□□□□▪▪▪▪▪▪▪▪□□□□□▪□□□□□□□□□□□□□▪▪▪	WA I	1592	1	4.3
Mafric	L6	ND	Undel	Del	▪▪▪▪▪▪□□□▪▪▪▪▪□▪▪▪▪▪▪▪▪▪▪▪▪▪▪▪▪▪▪▪▪▪▪▪□▪▪▪▪	WA 2	324	2	8.8
Mafric	L6	ND	Undel	Del	▪▪▪▪▪▪□□□▪▪▪▪▪▪▪▪▪▪▪▪▪▪▪▪▪▪▪▪▪▪▪▪▪▪▪▪▪□▪▪▪▪	WA 2	181	1	4.3
Mafric	L6	ND	Undel	Del	▪□▪▪▪▪□□□▪▪▪▪▪▪▪▪▪▪▪▪▪▪▪▪▪▪▪▪▪▪▪▪▪▪▪▪▪□▪▪▪▪	WA 2	318	1	4.3
Mafric	L6	ND	Undel	Del	▪▪▪▪▪▪▪▪▪□▪▪▪□□▪▪▪▪▪▪▪▪▪▪▪▪▪▪▪▪▪▪▪▪▪▪▪□▪▪▪▪	WA 2	Orphan	1	4.3

a Sub-lineage as defined by the MIRU-VNTRplus database, Undel = not deleted, Del = deleted, ND = Not done.

Associations between particular MTBC lineages and human ethnicities have been observed before. For example, in San Francisco, Lineage 1, 2 and 4 were strongly associated with Filipino, Chinese, and “white” ethnicities, respectively [11]. More recently, Hui ethnicity was found to be associated with the Beijing family of MTBC in China [35]. While social “cohesion” is likely to restrict intermingling between individuals belonging to different ethnic groups and thus transmission of MTBC between these groups, biological factors could also play a role in the association between different MTBC genotypes and human populations. Self-defined ethnicity has been shown to be a reliable proxy for human ancestry [36], and human genetic diversity has been linked to an increased or reduced susceptibility to TB [37]. Importantly, recent studies indicate that human genetic susceptibility to TB is further influenced by the MTBC genotype [10]. In particular, studies have reported human genetic polymorphisms that influence the susceptibility to TB caused by *M. africanum* but not *M. tuberculosis* sensu stricto or *vice versa* [38]. For example, a study performed in Ghana reported a human polymorphism in 5-lipoxygenase (ALOX5) associated with increased TB risk [39]. Stratification by MTBC lineage revealed that this association was mainly driven by *M. africanum* indicating that this human polymorphism increases the risk of TB in a MTBC lineage-specific matter. ALOX5 is involved in the synthesis of leukotrienes and lipoxins, which are important mediators of the inflammatory response [39]. Conversely, a human polymorphism reported recently in the Mannose Binding Lectin (MBL) was associated with protection against TB caused by *M. africanum* but not *M. tuberculosis* sensu stricto [40]. Moreover, this latter study also found that *M. africanum* bound human recombinant MBL more efficiently, perhaps leading to an improved uptake of *M. africanum* by macrophages and selection of deficient MBL variants among human populations exposed to *M. africanum* [40].

Our study has several limitations. First, data on HIV co-infection was not available. This might have influenced our results on the patient characteristics associated with *M. africanum*. Secondly, this study was not population-based as patients were recruited only at three government hospitals. Hence, some degree of selection bias cannot be excluded.

In conclusion, our study provides novel insights into the interaction between environmental, host and pathogen variability in human TB. In particular, the observed association between *M. africanum* and Ewe patient ethnicity suggests a possible explanation for the geographical restriction of *M. africanum* to parts of West Africa. Our findings also highlight the need to consider this variability in the development of new tools and strategies to control TB.

## Supporting Information

S1 Fig **Geographical distribution of** ***M. africanum*** **lineages by patient ethnic group.** Each dot stands for a single isolate and patient place of residence.
(PDF)

S1 Checklist **STROBE checklist.**
(DOCX)
