# Supplementary figures and images for: Mycobacterium africanum Is Associated with Patient Ethnicity in Ghana
Source: PLoS Negl Trop Dis. 2015 Jan 8;9(1):e3370. doi: 10.1371/journal.pntd.0003370 (PMC4287525; doi:10.1371/journal.pntd.0003370)

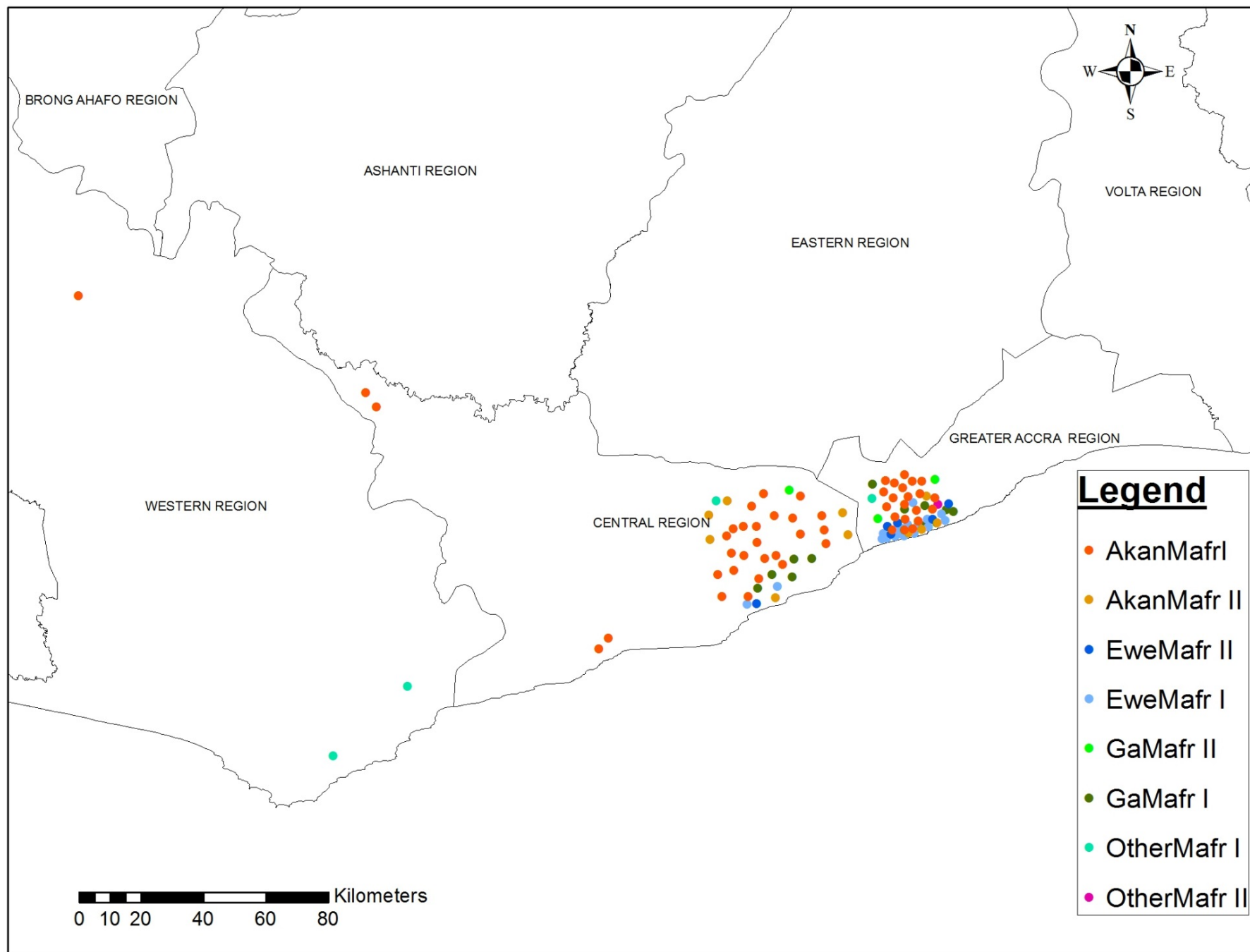

Supplement: S1 Fig — Geographical distribution of M. africanum lineages by patient ethnic group. Each dot stands for a single isolate and patient place of residence. (PDF) [file pntd.0003370.s001.pdf]
