# Supplementary material for: Mycobacterium africanum Is Associated with Patient Ethnicity in Ghana
Source: PLoS Negl Trop Dis. 2015 Jan 8;9(1):e3370. doi: 10.1371/journal.pntd.0003370 (PMC4287525; doi:10.1371/journal.pntd.0003370)
Supplement: S1 Checklist — STROBE checklist. (DOCX) [file pntd.0003370.s002.docx]

STROBE Statement—checklist of items that should be included in reports of observational studies

|  | Item No. | Recommendation | Page  No. | Relevant text from manuscript |
| --- | --- | --- | --- | --- |
| **Title and abstract** | 1 | (*a*) Indicate the study’s design with a commonly used term in the title or the abstract | 2 | … “To address this possibility, we performed a retrospective molecular epidemiological study of MTBC in Southern Ghana.” … |
|  |  | (*b*) Provide in the abstract an informative and balanced summary of what was done and what was found | 2 | … “Here we genotyped 613 MTBC clinical isolates from Ghana and searched for associations between the different phylogenetic lineages of MTBC and patient variables. We found *M. africanum* was  significantly more common in tuberculosis patients belonging to the Ewe ethnic group).” … |
| Introduction | | | |  |
| Background/rationale | 2 | Explain the scientific background and rationale for the investigation being reported | 5 | … “*M. africanum* is highly restricted to West-Africa for reasons unknown. One possibility could be that M. africanum has adapted to particular human populations in that region of the world.” … |
| Objectives | 3 | State specific objectives, including any pre specified hypotheses | 5 | … “combined bacterial genotyping with detailed demographic and epidemiological patient data and sought for associations between host factors and the main MTBC lineages prevailing in Ghana.” … |
| Methods | | | |  |
| Study design | 4 | Present key elements of study design early in the paper | 6 | … “The patients were recruited before treatment initiation from five main health facilities; ” … |
| Setting | 5 | Describe the setting, locations, and relevant dates, including periods of recruitment, exposure, follow-up, and data collection | 6 | … “The study was conducted from July 2007 to August 2011. The patients were recruited before treatment initiation from five main health facilities; Korle-Bu Teaching Hospital in the Greater Accra region, Agona Swedru Government Municipal Hospital, Winneba Government Hospital, St Gregory Catholic Clinic from the Central Region and Effia-Nkwanta Regional Hospital from Western Region of Ghana. Information on age, sex, nationality, ethnicity, employment status, previous history of TB, crowding, substance abuse and duration of symptoms were obtained from the patients with a structured questionnaire; ” … |
| Participants | 6 | (*a*) *Cohort study*—Give the eligibility criteria, and the sources and methods of selection of participants. Describe methods of follow-up  *Case-control study*—Give the eligibility criteria, and the sources and methods of case ascertainment and control selection. Give the rationale for the choice of cases and controls  *Cross-sectional study*—Give the eligibility criteria, and the sources and methods of selection of participants | 6 | … “All patients were diagnosed as sputum AFB-positive pulmonary TB cases by microscopy.” … |
|  |  | (*b*) *Cohort study*—For matched studies, give matching criteria and number of exposed and unexposed  *Case-control study*—For matched studies, give matching criteria and the number of controls per case | N/A | N/A |
| Variables | 7 | Clearly define all outcomes, exposures, predictors, potential confounders, and effect modifiers. Give diagnostic criteria, if applicable | 6 | … “Information on age, sex, nationality, ethnicity, employment status, previous history of TB, crowding, substance abuse and duration of symptoms were obtained from the patients with a structured questionnaire.” … |
| Data sources/ measurement | 8* | For each variable of interest, give sources of data and details of methods of assessment (measurement). Describe comparability of assessment methods if there is more than one group | 6 | … “with a structured questionnaire” … |
| Bias | 9 | Describe any efforts to address potential sources of bias | N/A |  |
| Study size | 10 | Explain how the study size was arrived at | N/A | N/A |

Continued on next page

| Quantitative variables | 11 | Explain how quantitative variables were handled in the analyses. If applicable, describe which groupings were chosen and why | N/A | N/A |
| --- | --- | --- | --- | --- |
| Statistical methods | 12 | (*a*) Describe all statistical methods, including those used to control for confounding | 8 | … “Multivariable logistic regression models were used to compare patient characteristics associated with *M. africanum* compared to *M. tuberculosis* sensu stricto. All statistical analyses were performed in STATA 10.1.” … |
|  |  | (*b*) Describe any methods used to examine subgroups and interactions | N/A | N/A |
|  |  | (*c*) Explain how missing data were addressed | 6/7 | … “Patients with missing information or culture-negative status were excluded from analysis.” … |
|  |  | (*d*) *Cohort study*—If applicable, explain how loss to follow-up was addressed  *Case-control study*—If applicable, explain how matching of cases and controls was addressed  *Cross-sectional study*—If applicable, describe analytical methods taking account of sampling strategy | N/A | N/A |
|  |  | (*e*) Describe any sensitivity analyses | N/A | N/A |
| Results | | | | |
| Participants | 13* | (a) Report numbers of individuals at each stage of study—eg numbers potentially eligible, examined for eligibility, confirmed eligible, included in the study, completing follow-up, and analysed | N/A | N/A |
|  |  | (b) Give reasons for non-participation at each stage | N/A | N/A |
|  |  | (c) Consider use of a flow diagram | N/A | N/A |
| Descriptive data | 14* | (a) Give characteristics of study participants (eg demographic, clinical, social) and information on exposures and potential confounders | 9 | … “Age of patients ranged from 8 to 77 years with a median age of 35 years (Table 1). Overall, 208/622 (33.4 %) were females with median age of 33 years; the remaining 414 (66.6%) were males with a median age of 36.” … |
|  |  | (b) Indicate number of participants with missing data for each variable of interest | 6/7 | … “Patients with missing information or culture-negative status were excluded from analysis.” … |
|  |  | (c) *Cohort study*—Summarise follow-up time (eg, average and total amount) |  |  |
| Outcome data | 15* | *Cohort study*—Report numbers of outcome events or summary measures over time |  |  |
|  |  | *Case-control study—*Report numbers in each exposure category, or summary measures of exposure |  |  |
|  |  | *Cross-sectional study—*Report numbers of outcome events or summary measures | N/A | N/A |
| Main results | 16 | (*a*) Give unadjusted estimates and, if applicable, confounder-adjusted estimates and their precision (eg, 95% confidence interval). Make clear which confounders were adjusted for and why they were included | 21/Table 3 | Ewe ethnicity were significantly more likely to present with TB caused by *M. africanum* as opposed to *M. tuberculosis* sensu stricto irrespective of their place of residence (odds ratio (OR) = 2.91 95% confidence interval (CI)**:** 1.65-5.141.  This association was independent from other risk factors.” … |
|  |  | (*b*) Report category boundaries when continuous variables were categorized | 21 / Table 1 | … “Yrs 08-25 Yrs 26-40 Yrs 41-77” … |
|  |  | (*c*) If relevant, consider translating estimates of relative risk into absolute risk for a meaningful time period | N/A | N/A |

Continued on next page

| Other analyses | 17 | Report other analyses done—eg analyses of subgroups and interactions, and sensitivity analyses | 10-11 | … “we performed a stratified analysis by lineage”… |
| --- | --- | --- | --- | --- |
| Discussion | | | | |
| Key results | 18 | Summarise key results with reference to study objectives | 12 | … “Our retrospective molecular epidemiological investigation of MTBC clinical isolates from Southern Ghana revealed that i) the Cameroon sub-lineage of Lineage 4 is the dominant cause of human TB in this region, ii) 17.1% of human TB is caused by M. africanum, iii) TB patients infected with M. africanum were more likely to smoke, and iv) to belong to the Ewe ethnic group.” … |
| Limitations | 19 | Discuss limitations of the study, taking into account sources of potential bias or imprecision. Discuss both direction and magnitude of any potential bias | 14 | … “This might have influenced our results on the patient characteristics associated with M. africanum. Secondly, this study was not population-based as patients were recruited only at three government hospitals. Hence, some degree of selection bias cannot be excluded.” … |
| Interpretation | 20 | Give a cautious overall interpretation of results considering objectives, limitations, multiplicity of analyses, results from similar studies, and other relevant evidence | 14 | … “In particular, the observed association between M. africanum and Ewe patient ethnicity suggests a possible explanation for the geographical restriction of M. africanum to parts of West Africa.” … |
| Generalisability | 21 | Discuss the generalisability (external validity) of the study results | 13 | … “Associations between particular MTBC lineages and human ethnicities have been observed before ” … |
| Other information | |  | | |
| Funding | 22 | Give the source of funding and the role of the funders for the present study and, if applicable, for the original study on which the present article is based | online | … “This study was supported by the Leverhulme-Royal Society Africa Award (grant AA080019 to DYM and SG), the National Tuberculosis Program Ghana, and the Swiss National Science Foundation (PP00P3_150750). Funders had no role in study design, data collection and analysis, decision to publish, or preparation of the manuscript ” … |

*Give information separately for cases and controls in case-control studies and, if applicable, for exposed and unexposed groups in cohort and cross-sectional studies.

**Note:** An Explanation and Elaboration article discusses each checklist item and gives methodological background and published examples of transparent reporting. The STROBE checklist is best used in conjunction with this article (freely available on the Web sites of PLoS Medicine at http://www.plosmedicine.org/, Annals of Internal Medicine at http://www.annals.org/, and Epidemiology at http://www.epidem.com/). Information on the STROBE Initiative is available at www.strobe-statement.org.
